# Supplementary material for: A silent Kv channel subunit shapes PV neuron action potential waveform and short-term synaptic plasticity during high-frequency firing
Source: bioRxiv. 2025 Nov 7:2025.11.06.686832. Preprint. [Version 1] doi: 10.1101/2025.11.06.686832 (PMC12637593; doi:10.1101/2025.11.06.686832)
Supplement: Supplementary file 1 [file NIHPP2025.11.06.686832V1-supplement-1.pdf]

bioRxiv preprint doi: https://doi.org/10.1101/2025.11.06.686832; this version posted November 7, 2025. The copyright holder for this preprint (which was not certified by peer review) is the author/funder, who has granted bioRxiv a license to display the preprint in perpetuity. It is made available under aCC-BY 4.0 International license.

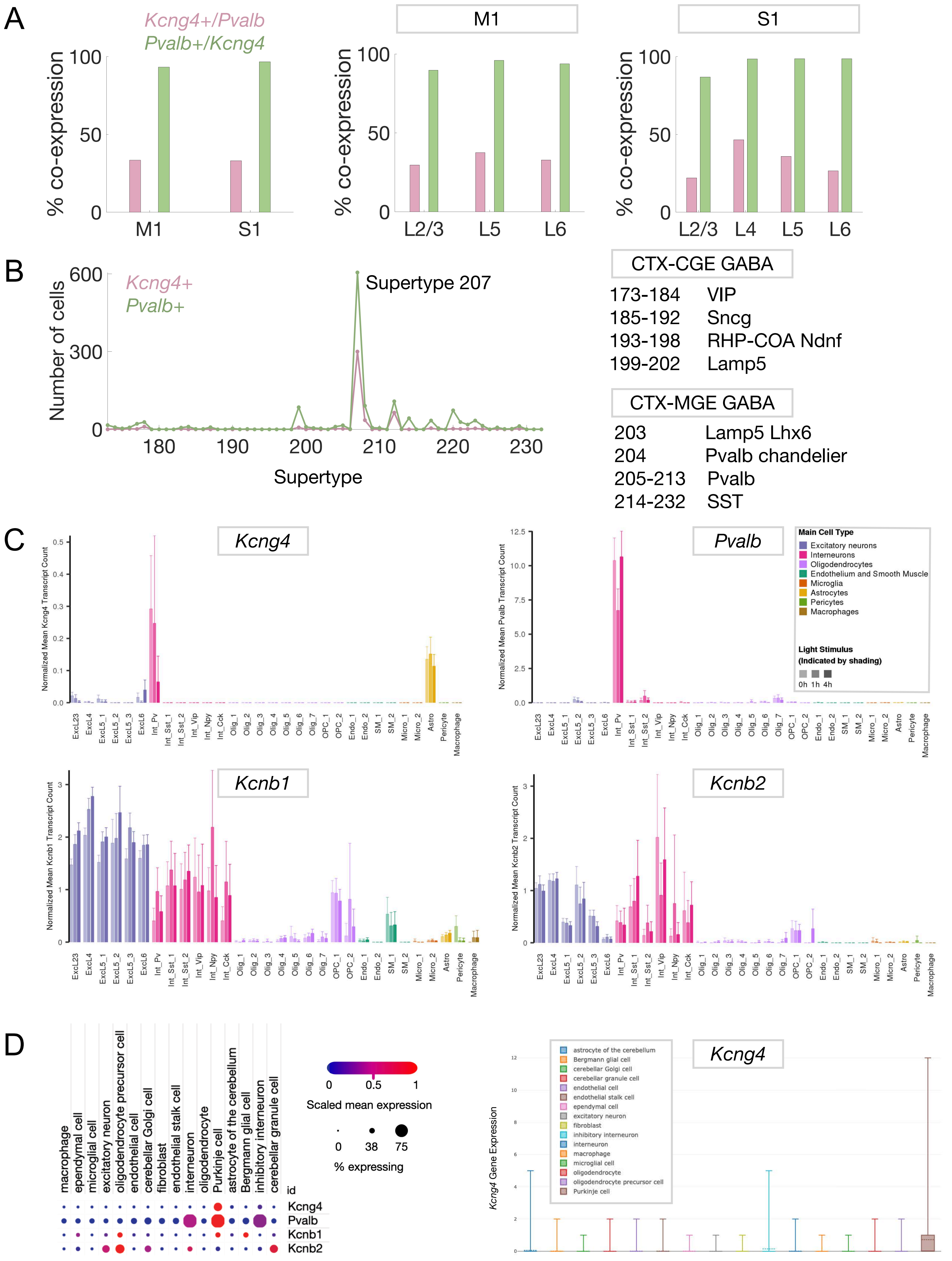

bioRxiv preprint doi: https://doi.org/10.1101/2025.11.06.686832; this version posted November 7, 2025. The copyright holder for this preprint (which was not certified by peer review) is the author/funder, who has granted bioRxiv a license to display the preprint in perpetuity. It is made available under aCC-BY 4.0 International license.

Fig. S1. Co-expression and cell-type-specificity of *Kcng4* and *Pvalb* across spatial and single-cell transcriptomic datasets.
A, Quantification of cells co-expressing *Kcng4* and *Pvalb* in adult mouse cortex was performed using Multiplexed Error-Robust Fluorescence *In Situ* Hybridization (MERFISH) data from the Allen Brain Cell (ABC) Atlas (Zhuang-ABCA-1, MERFISH whole brain coronal 1), accessed via the Brain Knowledge Platform (10). Percentage of co-expressing cells in M1 and S1, labeling as in **Fig. 1E**.
B, Number of *Kcng4*- and *Pvalb*-expressing cells in S1 across PV neuron supertypes, as classified by the ABC Atlas (*left*). Legend detailing inhibitory subtypes (*right*). Abbreviations: CTX, cortex; CGE, caudal ganglionic eminence; MGE, medial ganglionic eminence; GABA, GABAergic neuron; VIP, vasoactive intestinal peptide; Sncg, synuclein, gamma; RHP, retrohippocampal region; COA, cortical amygdalar area; Ndnf, neuron-derived neurotrophic factor; Lamp5, lysosomal-associated membrane protein family, member 5; Lhx6, LIM homeobox protein 6; Pvalb, parvalbumin; SST, somatostatin.
C, Normalized transcript counts for *Kcng4*, *Pvalb*, *Kcnb1*, and *Kcnb2* across cell types in mouse adult visual cortex from single-cell RNA sequencing data (3) accessed via an interactive gene expression database.
D, Gene expression for *Kcng4*, *Pvalb*, *Kcnb1*, and *Kcnb2* across cell types in mouse adult cerebellar cortex from single-nuclei RNA sequencing data (6) with a detailed visualization of *Kcng4* gene expression, accessed via the interactive Single Cell Portal.

bioRxiv preprint doi: https://doi.org/10.1101/2025.11.06.686832; this version posted November 7, 2025. The copyright holder for this preprint (which was not certified by peer review) is the author/funder, who has granted bioRxiv a license to display the preprint in perpetuity. It is made available under aCC-BY 4.0 International license.

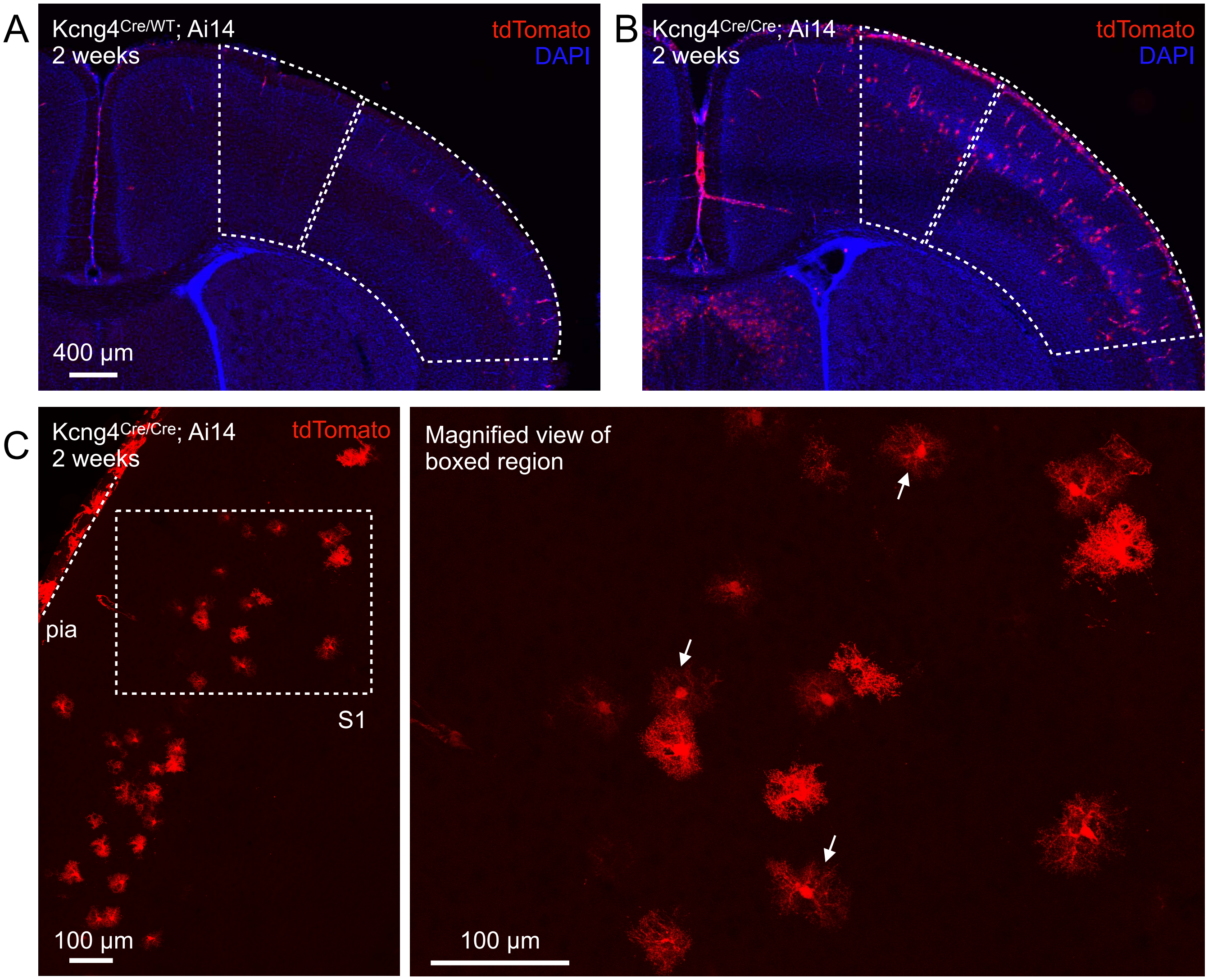

bioRxiv preprint doi: https://doi.org/10.1101/2025.11.06.686832; this version posted November 7, 2025. The copyright holder for this preprint (which was not certified by peer review) is the author/funder, who has granted bioRxiv a license to display the preprint in perpetuity. It is made available under aCC-BY 4.0 International license.

Fig. S2. Kcng4^{Cre} activation in juvenile mice.
A-B, Representative images showing tdTomato fluorescence in M1 and S1 for heterozygous (Kcng4$^{Cre/WT}$; Ai14) **(A)** and homozygous (Kcng4$^{Cre/Cre}$; Ai14) **(B)** mice at 2 weeks, corresponding to the regions outlined in **Fig. 2A**.
C, Higher magnification images illustrating the distribution of tdTomato-positive cells in S1 of a homozygous (Kcng4$^{Cre/Cre}$; Ai14) mouse. Magnified view (*right*) of the boxed region (*left*) marked by dashed white lines. Solid white arrows highlight example cells exhibiting astrocytic morphology (*right*).

bioRxiv preprint doi: https://doi.org/10.1101/2025.11.06.686832; this version posted November 7, 2025. The copyright holder for this preprint (which was not certified by peer review) is the author/funder, who has granted bioRxiv a license to display the preprint in perpetuity. It is made available under aCC-BY 4.0 International license.

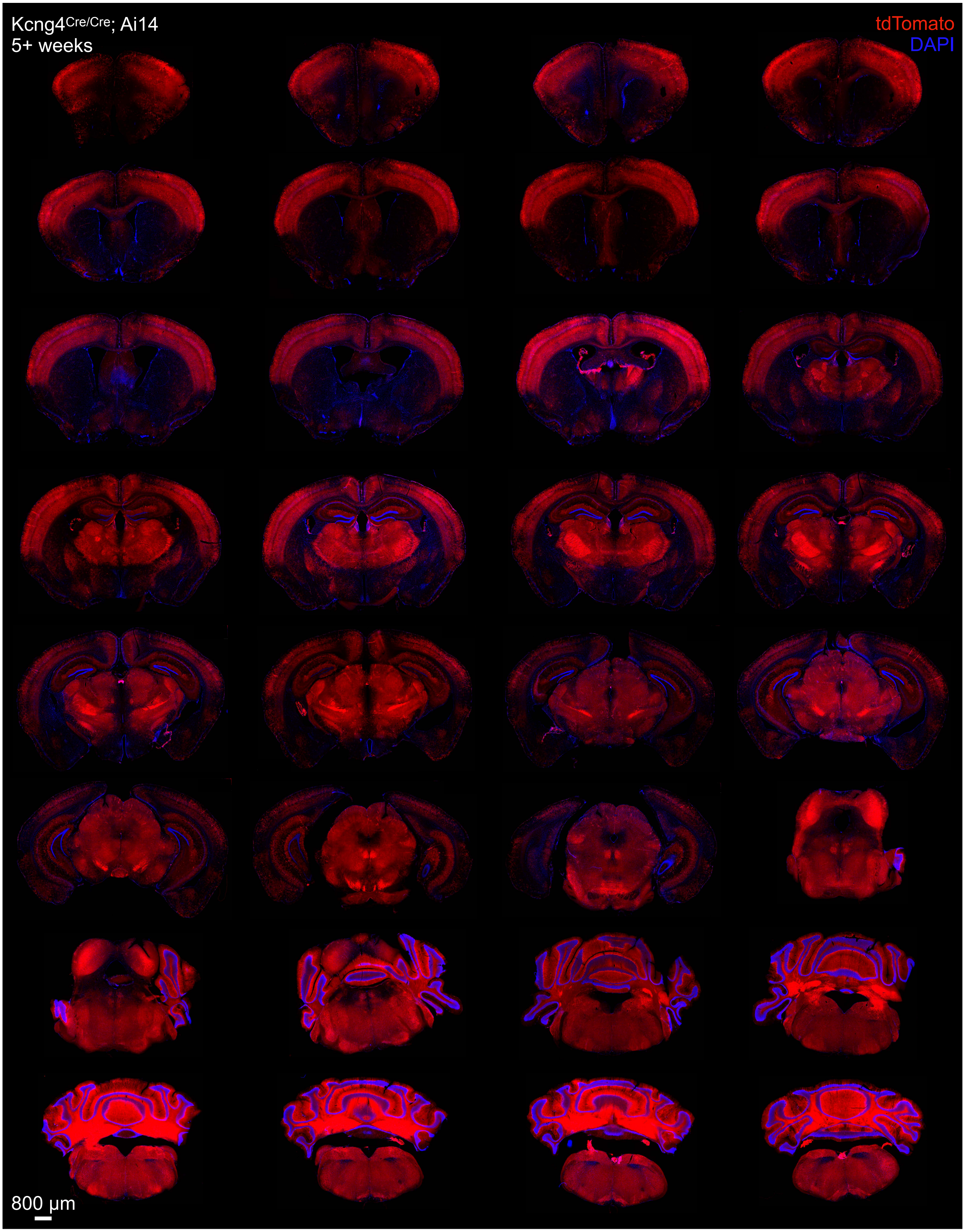

bioRxiv preprint doi: https://doi.org/10.1101/2025.11.06.686832; this version posted November 7, 2025. The copyright holder for this preprint (which was not certified by peer review) is the author/funder, who has granted bioRxiv a license to display the preprint in perpetuity. It is made available under aCC-BY 4.0 International license.

Fig. S3. Brain-wide Kcng4^{Cre} activation in adult mice.
Representative images showing tdTomato fluorescence in a homozygous (Kcng4$^{Cre/Cre}$; Ai14) mouse at 5+ weeks across various brain areas, arranged anterior to posterior.

bioRxiv preprint doi: https://doi.org/10.1101/2025.11.06.686832; this version posted November 7, 2025. The copyright holder for this preprint (which was not certified by peer review) is the author/funder, who has granted bioRxiv a license to display the preprint in perpetuity. It is made available under aCC-BY 4.0 International license.

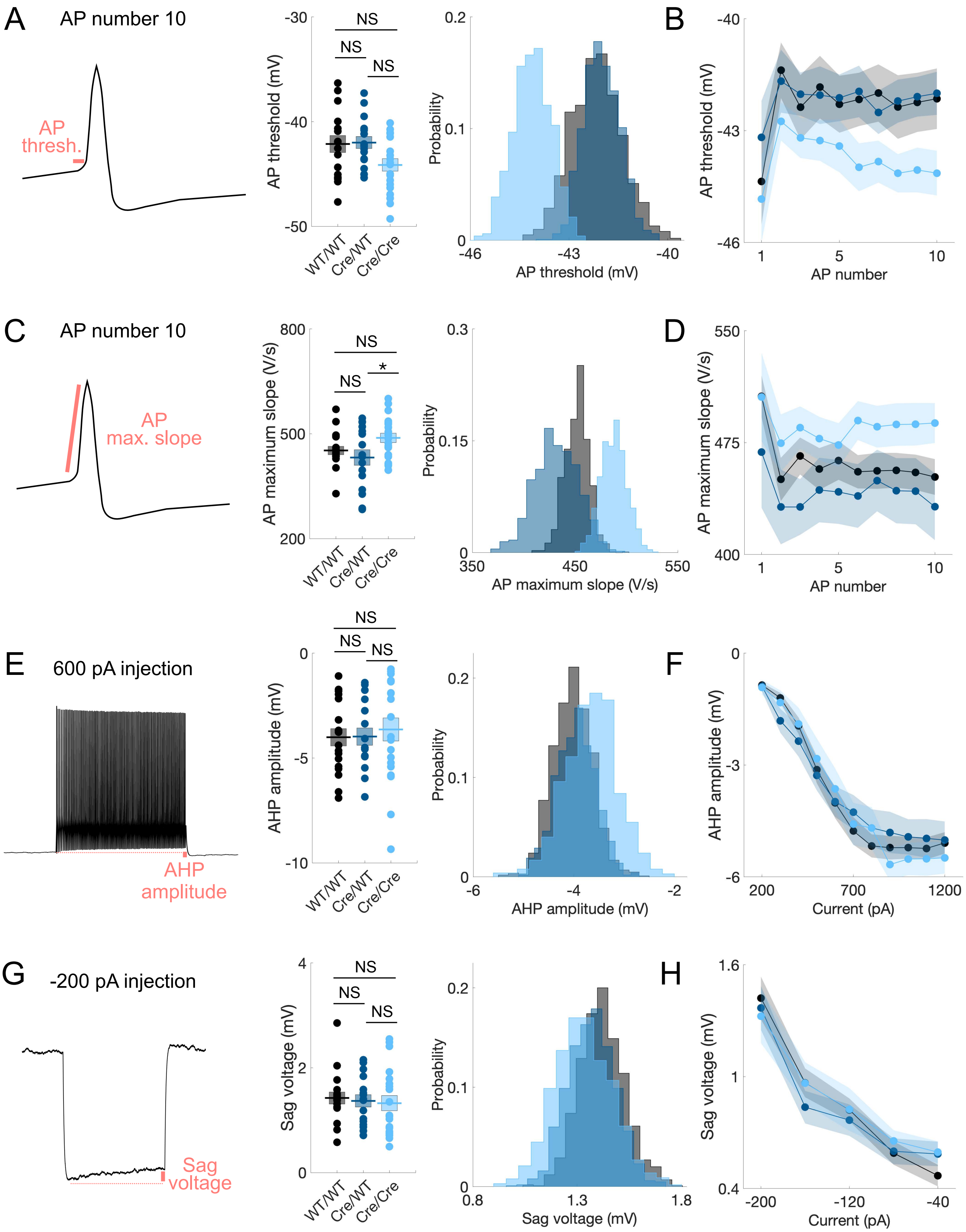

bioRxiv preprint doi: https://doi.org/10.1101/2025.11.06.686832; this version posted November 7, 2025. The copyright holder for this preprint (which was not certified by peer review) is the author/funder, who has granted bioRxiv a license to display the preprint in perpetuity. It is made available under aCC-BY 4.0 International license.

Fig. S4. Additional intrinsic properties of PV neurons following Kv6.4 deletion.
A, Schematic of AP threshold (*left*), group means (mV, *center*), and bootstrapped distribution of the means (*right*) for the 10th AP, as described in **Fig. 3**.
B, Mean values across the first 10 APs (mV).
C-H, As in **A** and **B** for AP maximum slope (V/s) (**C-D**), afterhyperpolarization (AHP; mV) amplitude (**E-F**), and sag voltage (mV) (**G-H**).
AHP amplitude was measured from a current injection of 600 pA (**E**) and across stepwise current injections from 200 to 1200 pA (**F**). AHP amplitude was defined as the difference between the resting membrane potential and the most negative voltage following current injection. Sag voltage was measured from a current injection of -200 pA (**G**) and across hyperpolarizing steps from -200 to -40 pA (**H**). Sag voltage was defined as the difference between the steady-state voltage and peak hyperpolarization during hyperpolarizing current injections.
Mean ± SEM displayed. * $P < 0.05$; NS: not statistically significant. One-way ANOVA (Tukey-Kramer post hoc multiple comparisons). Bootstrapping (1,000 iterations) was used to visualize sample mean distributions.

bioRxiv preprint doi: https://doi.org/10.1101/2025.11.06.686832; this version posted November 7, 2025. The copyright holder for this preprint (which was not certified by peer review) is the author/funder, who has granted bioRxiv a license to display the preprint in perpetuity. It is made available under aCC-BY 4.0 International license.

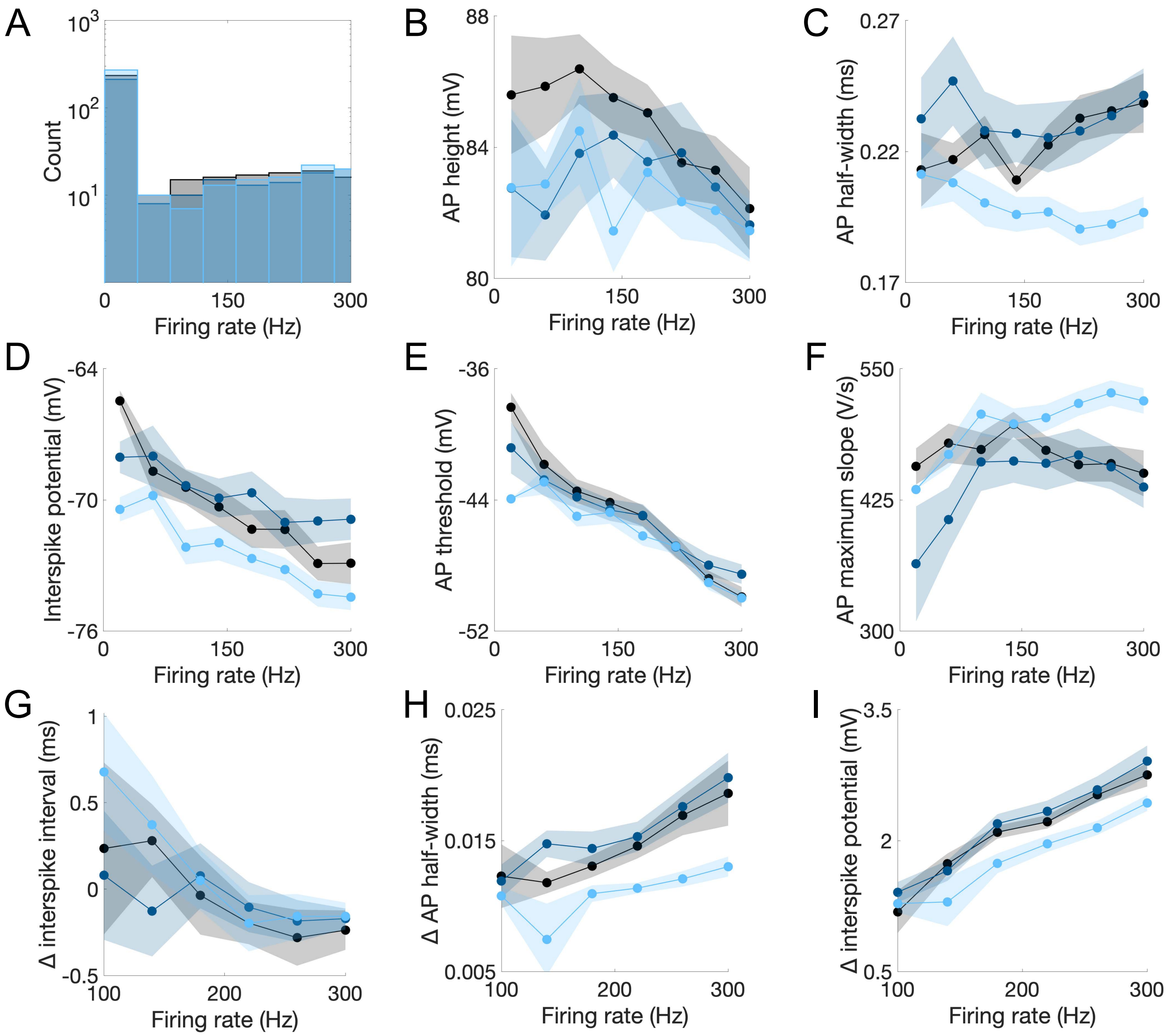

bioRxiv preprint doi: https://doi.org/10.1101/2025.11.06.686832; this version posted November 7, 2025. The copyright holder for this preprint (which was not certified by peer review) is the author/funder, who has granted bioRxiv a license to display the preprint in perpetuity. It is made available under aCC-BY 4.0 International license.

Fig. S5. Extended analysis of PV intrinsic properties following Kv6.4 deletion.
A, Histogram of firing rate (Hz) distribution (within 300 Hz) for all recordings, independent of current injection, across groups described in **Fig. 3**.
B, AP height (mV) across firing rate for all recordings.
C-F, As in **B** for AP half-width (ms) (**C**), interspike potential (mV) (**D**), AP threshold (mV) (**E**), and AP maximum slope (V/s) (**F**).
G, Change (Δ) in interspike interval (ISI; ms), defined as $ISI_{49\text{-}50} – ISI_{2\text{-}3}$, across firing rate in recordings containing ≥50 APs.
H-I, As in **G**, for Δ AP half-width (ms), defined as AP_{50} half-width – AP_2 half-width (**H**), and Δ interspike potential (mV), defined as AP_{50} interspike potential – AP_2 interspike potential (**I**).
Mean ± SEM displayed.

bioRxiv preprint doi: https://doi.org/10.1101/2025.11.06.686832; this version posted November 7, 2025. The copyright holder for this preprint (which was not certified by peer review) is the author/funder, who has granted bioRxiv a license to display the preprint in perpetuity. It is made available under aCC-BY 4.0 International license.

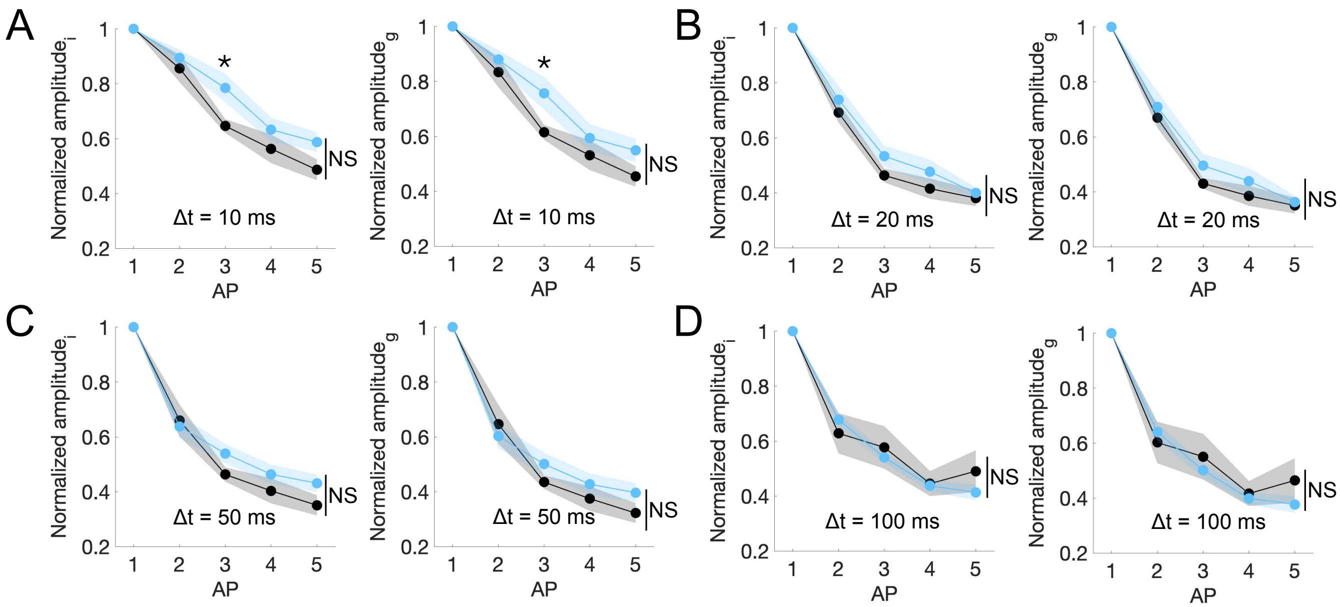

bioRxiv preprint doi: https://doi.org/10.1101/2025.11.06.686832; this version posted November 7, 2025. The copyright holder for this preprint (which was not certified by peer review) is the author/funder, who has granted bioRxiv a license to display the preprint in perpetuity. It is made available under aCC-BY 4.0 International license.

Fig. S6. Paired pulse depression at PV-PYR synapses across varying interstimulus intervals following Kv6.4 deletion.
A-D, Amplitude of inhibitory current (i; *left*, pA) and conductance (g; *right*, nS) across groups, normalized to response from the first AP, during trains of five presynaptic action potentials, as described in **Fig. 4**. Trains were delivered with an ISI (Δt) of 10 ms (**A**), 20 ms (**B**), 50 ms (**C**), and 100 ms (**D**).
Mean ± SEM displayed. * $P < 0.05$; NS: not statistically significant. Unpaired two-tailed t-tests or repeated-measures ANOVA (Greenhouse-Geisser corrected).

bioRxiv preprint doi: https://doi.org/10.1101/2025.11.06.686832; this version posted November 7, 2025. The copyright holder for this preprint (which was not certified by peer review) is the author/funder, who has granted bioRxiv a license to display the preprint in perpetuity. It is made available under aCC-BY 4.0 International license.

Datasets

Dataset S1 (separate file). Raw data and summary data for Fig. 1 and Fig. S1.

Dataset S2 (separate file). Summary data for Fig. 3 and Fig. S4.

Dataset S3 (separate file). Summary data for Fig. 4 and Fig. S6.
